# Idiopathic Tetanus

**Published:** 1883-04

**Authors:** James Rutherford

**Affiliations:** Babylon, Long Island


### III.—IDIOPATHIC TETANUS.

BY JAMES RUTHERFORD.

A stallion, aged four years, had been out to pasture for the whole summer. While roaming at large had appeared perfectly well. He was removed from the paddcock in the latter part of September and taken to a blacksmith to be shod. During the operation the animal was restless, and after the application of the shoes and when brought out to the road it was noticed that there was stiffness of the limbs and a staggery gait, or in the words of the groom, "He did not handle himself well."

During the following night he was attacked by marked symptoms of convulsions, which were tetanic in character. When I first saw the horse, which was the following morning, he had a stiff, straddling gait, increased respiratory movements, nostrils dilated, internal strabismus with twitching of the eyeballs. Slight noises seemed to agravate the spasms and consequently caused an irregular occurrence of the muscular contractions. The movements of the tongue and jaw were stiffened and later the maxilla became firmly locked.

The pulse was not markedly accelerated except during the spasm, when it would run up to 80 or 100 per minute.

The abdominal muscles were firmly contracted, the belly small and hard.

All the symptoms became more intense upon the second day. The bowels were constipated and the urine scanty during the whole progress of the disease.

The patient died at the end of forty-eight hours suffering from reflex convulsions.

*Treatment.*—As it was impossible for the horse to swallow I resorted to the hypodermic and rectal methods of administration, and gave Gelsemium ʒi every four hours, and Chloral Hydrate in Sol. ʒii every four hours per rectum. By the continued use of these remedies the spasms became less pronounced. The trismus, however, continued in a mild degree, but later on he had a very severe and prolonged spasm, which finally ended in death.

*Necropsy.*—Rigor mortis marked. No appreciable lesion could be found in any part of the body. All the internal organs normal. Body well supplied with adipose tissue.

A careful examination was made of the feet, but there was no apparent irregular portion and no point of injury by the nails of the recently applied shoes.

From the commencement, this case appeared to be idiopathic tetanus?

The case occurred on Long Island in a section of country where the disease is often endemic, and is chiefly noteworthy from the fact that it is usually developed in apparently healthy and young animals and without apparent cause, and that it runs its course rapidly and terminates fatally.

Horses are said to be more subject to the various forms of tetanus than any other domesticated animal. A number of cases have been reported from various sections in this vecinity during the last few months.

As yet we cannot boast of knowing very much of its pathology or etiology. Treatment seems of little avail. Perhaps some of our young and rising veterinarians may have it fall to their lot, even in the near future, to throw more light upon this obscure and imperfectly understood malady which now so often proves itself fatal to life.

*Babylon, Long Island,* DEC. 1882.

[These cases are undoubtedly a class which deserve careful and thoughtful consideration by the rising veterinary students and practitioners. We should hardly be inclined to consider either case purely idiopathic. It is a well known fact in human surgery that trivial wounds of the extreme ends of the limbs are often the most prolific causes of tetanus and it seems to us that the same must be true in equine surgery. It should be remembered that within the horny box of the horse's foot there is a very sensitive lamina, which may have been injured and the cause of the disease. In both cases we are inclined to the opinion that in these cases, owing to the great restiveness of the animal, the shoes were not properly applied, the barings changed and the sensitive lumina injured and thus the tetanus produced. It is not necessary to have a misplaced nail as the exciting cause. This fact seems to be fairly well substantiated by the history of these two cases.

The striking similarity of these cases certainly furnishes material for thoughtful study and further careful observation. The question also arises, are stallions more subject to this form of tetanus than other animals?

Another point of interest in the first case is the diminuition in the quantity of the urine passed and suggests the possibility of some renial complication. We are not, however, informed in reference to any further examination of the same, and as we have often had occasion to remark, veterinarians seem to neglect entirely this important branch of the medical science.

We feel it our duty to continually call attention to the importance of these urinary analysis until it becomes a part of every practitioner's examination. We should be pleased to hear from the experience of all practitioners and ask them to carefully note the point in reference to these attacks occurring after application of a new set of shoes.—EDS.]

